# Nonlocal topological insulators: Deterministic aperiodic arrays supporting localized topological states protected by nonlocal symmetries

**DOI:** 10.1073/pnas.2100691118

**Published:** 2021-08-19

**Authors:** Kai Chen, Matthew Weiner, Mengyao Li, Xiang Ni, Andrea Alù, Alexander B. Khanikaev

**Affiliations:** ^a^Department of Electrical Engineering, Grove School of Engineering, City College of the City University of New York, New York, NY 10031;; ^b^Physics Program, Graduate Center of the City University of New York, New York, NY 10016;; ^c^Photonics Initiative, Advanced Science Research Center, City University of New York, New York, NY 10031

**Keywords:** topological materials, aperiodic systems, higher-order topology

## Abstract

The properties of topological systems are inherently tied to their dimensionality. Indeed, higher-dimensional periodic systems exhibit topological phases not shared by their lower-dimensional counterparts. On the other hand, aperiodic arrays in lower-dimensional systems (e.g., the Harper model) have been successfully employed to emulate higher-dimensional physics. This raises a general question on the possibility of extended topological classification in lower dimensions, and whether the topological invariants of higher-dimensional periodic systems may assume a different meaning in their lower-dimensional aperiodic counterparts. Here, we demonstrate that, indeed, for a topological system in higher dimensions one can construct a one-dimensional (1D) deterministic aperiodic counterpart which retains its spectrum and topological characteristics. We consider a four-dimensional (4D) quantized hexadecapole higher-order topological insulator (HOTI) which supports topological corner modes. We apply the Lanczos transformation and map it onto an equivalent deterministic aperiodic 1D array (DAA) emulating 4D HOTI in 1D. We observe topological zero-energy zero-dimensional (0D) states of the DAA—the direct counterparts of corner states in 4D HOTI and the hallmark of the multipole topological phase, which is meaningless in lower dimensions. To explain this paradox, we show that higher-dimension invariant, the multipole polarization, retains its quantization in the DAA, yet changes its meaning by becoming a nonlocal correlator in the 1D system. By introducing nonlocal topological phases of DAAs, our discovery opens a direction in topological physics. It also unveils opportunities to engineer topological states in aperiodic systems and paves the path to application of resonances associates with such states protected by nonlocal symmetries.

## Significance

A concept of nonlocal topological phases of DAAs introduced here establishes a different direction in topological physics and offers approaches to emulate higher-dimensional topology in lower-dimensional systems. Our study also unveils opportunities to engineer topologically protected states in aperiodic systems and paves the path to application of resonances associated with such states, whose robustness is ensured by nonlocal symmetries of DAAs. In particular, the possibility to engineer multiple localized resonances via dimensional reduction and their unique features, such as precise spectral properties stemming from their topological nature, offers remarkable opportunities for practical applications, from robust resonators to sensors and aperiodic topological lasers.

The dimensionality of a topological system plays a determining role in defining its symmetry classification and, therefore, the topological invariants that identify the topological phase specific to such a system (1–3). For instance, four-dimensional (4D) quantum Hall systems can exhibit a nonvanishing second-class Chern number that is not shared by systems with three or fewer dimensions, and hence it cannot be implemented in three physical dimensions without mapping to a lower-dimensional analog (4). However, already 3D topological materials imply challenging fabrication demands due to the complex structures of the lattices (5–9). This quest has been approached in two ways: 1) introducing synthetic dimensions or 2) mapping a higher-dimensional system onto its lower-dimensional counterpart. The first approach implements lattices with dimensions higher than the spatial dimensions by exploiting internal degrees of freedom, which could be spectral (10–18), temporal, or spatial in nature (19–23). The second approach, based on dimensional reduction and mapping onto a lower-dimensional system, has been proven quite fruitful too; as an example, the celebrated Harper–Hofstadter Hamiltonian has been recently emulated in reconfigurable quasi-periodic 1D resonant acoustic lattices (24, 25). The topological properties of quasiperiodic models via dimensional reduction have also been studied (26–28). In addition, the existence of boundary modes stemming from the second Chern class topological phase has been reported in photonics (4) and in an angled optical superlattice of ultracold bosonic atoms (29).

As we show here, the second approach, in addition to the possibility to emulate higher-dimensional physics in lower dimensions, establishes an approach to engineer novel nonlocal topological phases supporting localized topological states. We focus here on the recently introduced class of higher-order topological insulators (30, 31) and show that their lower-dimensional analogs are characterized by nonlocal symmetries which ensure the presence and protect the localized topological states. In recent years higher-order topological insulators (HOTIs) were successfully realized in 2D (32–39), and more recently 3D octupole topological states were implemented in 3D acoustic metamaterials (40, 41). The choice of HOTIs as an exemplary system is justified by the fact that their dimensionality defines the multiplicity of hosted topological boundary modes protected by higher-dimensional reflection and chiral symmetries.

In this work we apply dimensionality reduction and experimentally realize a 1D counterpart of 4D hexadecapolar HOTI (h-HOTI) in the form of a deterministic aperiodic array (DAA). To this aim, we apply Lanczos tridiagonalization introduced recently by Maczewsky et al. (42) which allows mapping of any multidimensional system onto aperiodic 1D tight-binding system with nearest-neighbor coupling. In their original work Maczewsky et al. introduced the mapping of a periodic higher-dimensional system onto a 1D tight-binding model (TBM) based on Lanczos transformation, and they demonstrated how introduction of a point defect leads to the localization in systems of different dimensionalities. Here, we apply Lanczos transformation to investigate topological systems, where, due to the bulk-boundary correspondence, the localization of topological boundary modes is caused by the nontrivial bulk topology of a higher-dimensional system. By this approach we map a prototypical 4D h-HOTI tight-binding system onto a 1D DAA with strictly local aperiodic coupling distribution. We note that the obtained aperiodic system is not and should not be confused with quasiperiodic systems which can exhibit rich topological physics on their own (26–28). The transformation of topological invariant of the original 4D system—the quadrupole polarization—yields a new 1D invariant, a topological correlator, with the same value as its 4D analogs, which ensures the presence of 16 zero-energy topological states localized in the bulk of DAA. By the special choice of the Lanczos transformation (42–45) we obtain one topological state localized the edge of our sample. Other corner states are distributed in the bulk of the array. All the states preserve their topological characteristics, being protected by nonlocal symmetries of 1D DAA originating in higher-dimensional symmetries of 4D h-HOTI.

The designed 1D DAA with nonlocal topological phase is then realized in an array of coupled acoustic resonators. The presence of localized topological states is directly observed by measuring local acoustic pressure field distribution in the array.

## Results

**Fig. 1.**
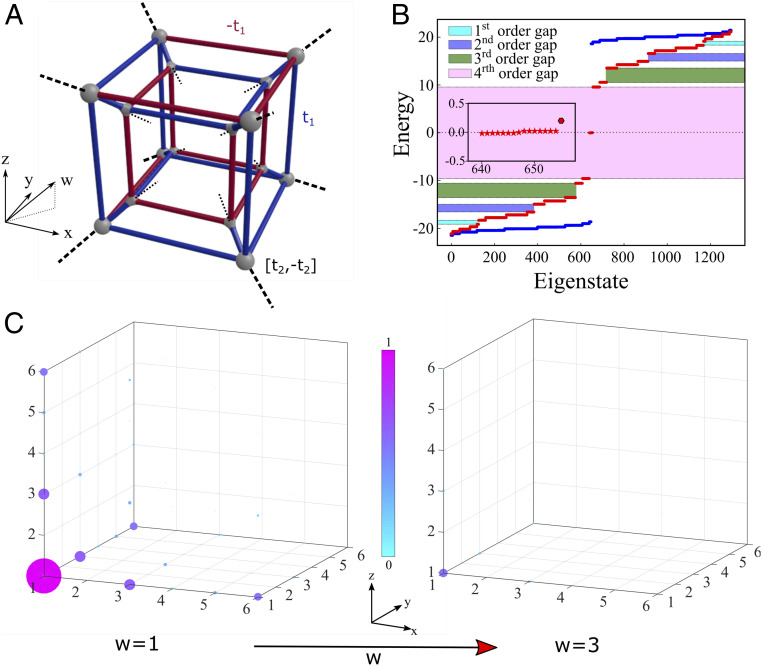
Unit cell, band spectra, and corner modes of 4D h-HOTI. (*A*) Schematic of the unit cell of a 4D higher-order topological insulator. The blue lines indicate positive hopping (zero phase) and the red lines indicate negative hopping (π phase), so that each hypersurface has net flux of π. The black dashed lines indicate intercell connections to the lattice (with phases of 0 and π). (*B*) Band spectra for the trivial (blue dots) hyperlattice corresponding to intracell hopping amplitude $t_{1}$ larger than intercell hopping $t_{2}$ ($t_{1}/t_{2}=10$). Band spectra for topologically nontrivial (red dots) higher-order phase with $t_{1}<t_{2}$ ($t_{2}/t_{1}=10$), $\delta =0.2t_{1}$. (*Inset*) A zoom-in confirming the presence of 16 corner modes. Colored regions indicate topological bandgaps of different order. The first-order gap is the gap between 4D bulk spectrum and 3D bulk spectrum, the second-order gap is the gap between 3D bulk spectrum and surface spectrum, the third-order gap is the gap between surface spectrum and edge spectrum, and the fourth-order gap is the gap between edge spectrum and corner spectrum. (*C*) Intensity distribution of corner mode across the lattice, showing the field decay and localization in all four dimensions. Each subplot shows the field distribution projected on the *xyz*-coordinate frame for specific values of w=1 and w=3 corresponding to the same sublattice. The corner mode shown corresponds to the energy eigenvalue indicated by the enlarged (hexagon-shaped) red point in *B*, *Inset*.

We start with the prototypical 4D h-HOTI described by a tight-binding Hamiltonian with nearest-neighbor coupling in 4D hypercubic unit cell, as schematically shown in Fig. 1*A*. In Bloch representation, the Hamiltonian can be expressed as

$${\hat{H}}_{4D}\left(k\right)=t_{2}\sin\left(k_{x}\right)\;{\hat{\Gamma }}_{3}+\left[t_{1}+t_{2}\cos\left(k_{x}\right)\right]{\hat{\Gamma }}_{4}+t_{2}\text{sin}\left(k_{y}\right)\;{\hat{\Gamma }}_{1}$$

$$+\left[t_{1}+t_{2}\cos\left(k_{y}\right)\right]{\hat{\Gamma }}_{2}+t_{2}\text{sin}\left(k_{z}\right)\;{\hat{\Gamma }}_{5}+\left[t_{1}+t_{2}\cos\left(k_{z}\right)\right]{\hat{\Gamma }}_{6}$$

$$+t_{2}\sin\left(k_{w}\right){\hat{\Gamma }}_{7}+\left[t_{1}+t_{2}\cos\left(k_{w}\right)\right]{\hat{\Gamma }}_{8},$$ [1]

where $t_{1}$ and $t_{2}$ are the nearest-neighbor intracell and intercell hopping amplitudes, respectively. In Eq. **1**, the matrices ${\hat{\Gamma }}_{i}=-\sigma_{3}⊗\sigma_{3}⊗\sigma_{2}⊗\sigma_{i}$ for *i* = 1,2,3, ${\hat{\Gamma }}_{4}=\sigma_{3}⊗\sigma_{3}⊗\sigma_{1}⊗\sigma_{0}$, ${\hat{\Gamma }}_{5}=\sigma_{3}⊗\sigma_{2}⊗\sigma_{0}⊗\sigma_{0}$, ${\hat{\Gamma }}_{6}=\sigma_{3}⊗\sigma_{1}⊗\sigma_{0}⊗\sigma_{0}$, ${\hat{\Gamma }}_{7}=\sigma_{1}⊗\sigma_{0}⊗\sigma_{0}⊗\sigma_{0}$, and${\hat{\Gamma }}_{8}=\sigma_{2}⊗\sigma_{0}⊗\sigma_{0}⊗\sigma_{0}$, $\sigma_{i=1,2,3}$ are Pauli matrices; $\sigma_{0}$ is the identity matrix and k is the Bloch wave number.

The energy spectrum found from the 4D TBM of a finite (3 × 3 × 3 × 3 × 16) h-HOTI lattice with π-flux through each 2D plaquette of the unit cell is shown in Fig. 1*B*. The blue points in Fig. 1*B* correspond to the energy spectrum of a topologically trivial phase, with intracell hopping amplitude exceeding the intercell hopping $t_{2}<t_{1}$, while the red dots show the energy spectrum of a topologically nontrivial HOTI phase, with $t_{2}>t_{1}$. In the latter case, the system of finite size in all four dimensions hosts 16 fourth-order topological corner states, due to the higher-order bulk-boundary correspondence (30, 31) (see more detailed discussion in *SI Appendix*, sections S1and S2). The intensity distribution of one of the corner modes within the h-HOTI, obtained from TBM, is shown in Fig. 1*C*, which confirms that this is the fourth-order state localized to the corner of the hyperlattice in all four (x,y,z,w) directions. In order to make the selected corner state better noticeable, we added a small on-site potential to the respective corner site ($\delta =0.2t_{1})$ to split this state from the rest of the corner modes.

We now map this finite 4D h-HOTI onto a 1D DAA of coupled resonators with aperiodic hopping amplitudes using the Lanczos transformation (42). The Lanczos transforms a general Hermitian matrix into a tridiagonal form and, therefore, allows for an immediate mapping onto a 1D TBM with an only-nearest-neighbor hopping distribution characterized by an inhomogeneous (aperiodic) profile. The Lanczos transformation is unitary, which ensures preserving spectrum and eigenstate orthogonality of the original Hamiltonian. This property guarantees that the final (tridiagonal) effective 1D Hamiltonian will still be Hermitian ${\hat{H}}_{1D}^{eff}={\hat{U}}_{L}{\hat{H}}_{4D}{\hat{U}}_{L}^{-1}=\underset{i}{\sum }\epsilon_{i}C_{i}^{+}C_{i}+\underset{\text{i}}{\sum }\tau_{i}C_{i}^{+}C_{i+1}+c.c$, where ${\hat{U}}_{L}$ is the Lanczos transformation operator and $\epsilon_{i}$ and $\tau_{i}$ are the effective on-site energy and hopping amplitudes in the 1D array, and that the number of degrees of freedom of the 4D Hamiltonian is preserved.

For convenience, we also use the fact that the Lanczos transformation can be performed with a specific site of choice (i.e., a component of the eigenvector) being fixed, known as an anchor site (42). Thus, any chosen site of the 1D array will have a one-to-one correspondence with one of the sites of the original higher-dimensional lattice, implying that a component of the eigenstate at a chosen site is fully preserved. This property can be used to make sure that at least one corner site in 4D is pinned (“anchored”) to the edge site (or any chosen bulk site, if needed) of the 1D array, implying that the corresponding corner state confined to this site in 4D will remain localized at the physical boundary of our aperiodic 1D lattice.

**Fig. 2.**
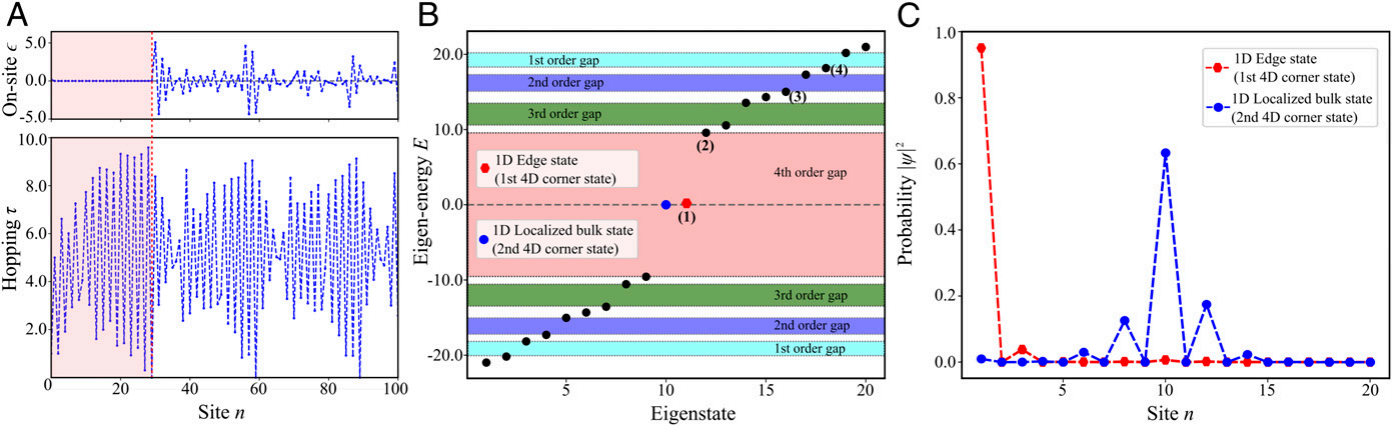
Band spectra and boundary mode of the truncated 1D resonator array mapped from 4D h-HOTI. (*A*) Hopping amplitudes and onsite energies (in the unit of *t*_1_) of the effective 1D model. (*B*) Band spectra of the effective 1D model truncated to 20 sites obtained using the TBM. The colored regions separate modes of different order and represent topological band gaps inherited from 4D h-HOTI. (*C*) Intensity distributions corresponding to the projected fourth-order topological corner mode in the 1D array, as found from tight-binding calculations.

The results of Lanczos transformation applied to the 4D Hamiltonian (Fig. 1*A*) are shown in Fig. 2*A*, where the first 100 hopping amplitudes $\tau_{n}$ and on-site energies $\epsilon_{n}$ of the effective aperiodic 1D model are plotted. The real-valued character of the 4D Hamiltonian (only phase of 0 and π for hopping $\tau_{n}$) ensures that the Lanczos operator ${\hat{U}}_{L}$ is an orthogonal matrix, yielding real values for onsite energies and real positive hopping amplitudes in the dimensionally reduced 1D DAA system. We numerically confirmed that the mapped 1D Hamiltonian has a spectrum identical to the one of the 4D HOTI, as expected, and that the anchored corner state appears pinned to the edge of the 1D system. Moreover, we confirmed that the inverse transformation of a 1D boundary state maps it onto corner states localized at the anchor site in 4D HOTI. It is worth mentioning that the unitary character of the transformation was enforced by implementing Lanczos biorthogonalization algorithm (45), which ensured numerical stability and one-to-one correspondence between the spectrum and eigenstates in 4D and 1D systems.

Interestingly, as the used transformation preserves only the anchor site, all other zero-energy 4D corner states appear in the bulk of the 1D lattice, but they have to remain spatially localized and topologically protected (a proof can be found in *SI Appendix*, section S3). However, since the number of higher-order boundary states (16 for the 4D case considered here) exceeds the number of boundaries of the projected 1D system (only two), most of the projected topological corner modes localize in the bulk of the 1D array. The proposed procedure thus not only offers an emulation of higher-order topological states in lower-dimensional systems but also unveils a new class of localized bulk resonances topologically protected by higher-dimensional symmetries of HOTIs. Indeed, the inspection of the eigenstates of the effective 1D system confirms that all other corner states of the original h-HOTI are zero-energy states which localize in the bulk of the DAA (see amplitude distribution of these corner modes in *SI Appendix*, section S4).

It is worth noting that the final Hamiltonian of the DAA obtained by the Lanczos transformation depends on the choice of the “trial” vector (which also fixes the anchor site), and thus there exists a family of different DAAs all corresponding to the same 4D HOTI. Member Hamiltonians of this family of DAAs, however, are topologically equivalent to one another and to the original higher-dimensional system as they all are characterized by the same topological invariant (see discussion and calculations below). Similarly, if an alternative tridiagonalization transformation is chosen, e.g., Householder transformation, it will produce another topologically equivalent DAA. A clear advantage of the Lanczos transformation, however, is in the possibility to pin the position of one of the topological corner states by choosing any desirable anchor site.

The emergence of a quantized multipole moment and corner states in the 4D h-HOTI is deeply related to the symmetries of the system, i.e., the presence of anticommuting reflection symmetries. Similarly, the “zero energy” of the corner states is ensured by the chiral symmetry $\hat{\Gamma }{\hat{H}}_{4D}\;{\hat{\Gamma }}^{-1}=-{\hat{H}}_{4D}$, which stems from the fact that in the h-HOTI the sites that belong to the same sublattice do not couple with each other. It is therefore clear that DAA must inherit the topological nature of its higher-dimensional prototype, while the emergence of the localized zero-energy states in 1D DAA further evidences the nontrivial topology of the system.

To prove this conjecture, we will consider the role of original symmetries of h-HOTI in the projected 1D DAA. We first consider the chiral symmetry of the system, which has matrix representation $\hat{\Gamma }=\sigma_{3}⊗\sigma_{3}⊗\sigma_{3}⊗\sigma_{0}$, and ensures the overall symmetry of the spectrum with respect to zero energy and “zero energy” of the topological corner states. The fact that this property is retained during the dimensional reduction implies that the chiral symmetry is retained as well, but in a new form expressed as ${\hat{U}}_{L}\hat{\Gamma }{\hat{U}}_{L}^{-1}$.

Due to its local nature (i.e., imposing constraint on the unit cell length scale), the chiral symmetry can be written in the diagonal form as ${\hat{\Gamma }}^{finite}=I_{N\times N}⊗\hat{\Gamma }$, where $I_{N\times N}$ is the N by N identity matrix and N is the number of degrees of freedom (unit cells). The Lanczos transformation changes this local character of the chiral symmetry by mixing different sites ${\hat{\Gamma }}_{1D}^{eff}={\hat{U}}_{L}{\hat{\Gamma }}^{finite}{\hat{U}}_{L}^{-1}$, thus producing the new nonlocal form of chiral symmetry ${\hat{\Gamma }}_{1D}^{eff}{\hat{H}}_{1D}^{eff}\;{\hat{\Gamma }}^{eff-1}=-{\hat{H}}_{1D}^{eff}$ in the 1D DAA (see *SI Appendix*, section S5 for details). This nonlocal chiral symmetry of the 1D Hamiltonian plays the same role as the original chiral symmetry for 4D h-HOTI and it ensures spectral symmetry of the modes of aperiodic 1D array with respect to zero energy. Such a nonlocal character of the symmetry operator ${\hat{\Gamma }}_{1D}^{eff}$ of DAA reflects the presence of correlations of parameters within the array (hopping amplitudes and on-site energies), which, in analogy with the conventional “local” chiral symmetry, will pin down the zero-dimensional (0D) topological states to the zero energy.

Similarly, the h-HOTI Hamiltonian (1) possesses reflection symmetries that anticommute with each other due to the flux of π in each of the plaquettes of the hypercubic lattice. The reflection symmetries result in a nonvanishing topological invariant, the quantized multipole moment of the bulk bands. Similar to the case of boundless periodic systems, when analysis is performed in Bloch (momentum) space (30, 31), in the case of finite systems, the topological invariant can be extracted from the respective eigenfunctions calculated in the real-space representation (46), the procedure which is customarily used in analysis of disordered and quasiperiodic systems (27, 28, 47–50). Nonetheless, even if the evaluation of multipole polarization is possible in the DAA, from a geometric point of view it is a meaningless quantity in 1D, and thus it should be understood and interpreted differently.

To confirm that the topological invariant retains its value in DAA, we evaluated its value for the finite system by applying a generalized theory which was developed for amorphous systems in Agarwala et al. (46). With the use of the eigenstates $|\alpha_{n}>$ and eigenenergies $E_{n}$ of the finite 4D h-HOTI system we define matrix $\hat{P}=\left\{|\alpha_{1}>,|\alpha_{2}>,\ldots ,|\alpha_{N_{0cc}}>\right\}$ of size $N\times N_{occ}$, where $N_{occ}$ indicates the number of occupied (or negative energy for half-filling) states. We then introduce a diagonal matrix operator $\hat{D}=diag\;\left\{\text{exp}\left[i2\pi \frac{f\left(r_{1}\right)}{N}\right],\text{exp}\;\left[i2\pi \frac{f\left(r_{2}\right)}{N}\right],\ldots \text{exp}\left[i2\pi \frac{f\left(r_{N}\right)}{N}\right]\right\}$, where for the h-HOTI $f\left(r_{k}\right)$ = $x_{k}y_{k}z_{k}w_{k}$ and the diagonal form indicates the local character of the hexadecapole moment and of the respective antireflection symmetry. The hexadecapole moment, the quantity capturing the topological properties of 4D h-HOTI, is then expressed as $h_{xyzw}=\left(\frac{1}{2\pi }Im\;Tr\;\left[\ln\left({\hat{P}}^{+}\hat{D}\hat{P}\right)\right]-\frac{n_{f}}{N}\;Tr\left[E\right]\right)modulo\;1$, where $n_{f}=\frac{1}{2}$ is the filling in the system and $E=diag\left\{f\left(\vec{r_{1}}\right),f\left(\vec{r_{2}}\right),\ldots f\left(\vec{r_{N}}\right)\right\}$ . We confirmed that for both open and periodic boundary conditions calculation of $h_{xyzw}$ yields the value 1/2.

Application of the Lanczos transformation to the above definition of a topological invariant allows us to obtain a 1D equivalent for the DAA system $h_{DAA}=\left(\frac{1}{2\pi }Im\;Tr\;\left[\ln\;\left({\tilde{P}}^{+}\;\tilde{D}\tilde{P}\right)\right]-\frac{n_{f}}{N}\;Tr\left[E\right]\right)modulo\;1$, where the matrix $\tilde{P}=\left\{|\tilde{\alpha_{1}}>,|\tilde{\alpha_{2}}>,\ldots ,|\tilde{\alpha_{N_{occ}}}>\right\}$ is constructed using occupied states of the 1D aperiodic array and $\tilde{D}={\hat{U}}_{L}\hat{D}{{\hat{U}}_{L}}^{+}$ is the transformed symmetry operator which acquires a nondiagonal form. In contrast to the case of diagonal from of the local symmetry operator $\hat{D}$, the presence of off-diagonal components in $\tilde{D}$ emphasizes the nonlocal character of correlations within DAA. These correlations, however, do originate from the local reflection symmetry in the original 4D h-HOTI and the resultant nonvanishing hexadecapole moment.

Even though the topological invariant loses its original geometric meaning, our calculations applied to the eigenstates of DAA confirm that it retains its quantized value of 1/2 due to the presence of such nonlocal correlations (*SI Appendix*, section S6). The same calculations for the trivial case show vanishing hexadecapole moment and lack of localized zero-energy states in both 4D HOTI and 1D DAA systems.

It is worth noting that the procedure of evaluation of the topological invariant based on real-space data used above can also be applied in experiments, which, however, would require complete information about amplitudes and phases in all the sites of experimental structure, which must be gathered for all low-frequency (with the frequencies below the topological bandgap) bulk states. On the other hand, an experimental observation of localized zero-energy states, and of the symmetric spectrum of all other modes with respect to zero energy, would already represent a direct evidence of nonlocal symmetries and of the topological nature of DAA.

To summarize, despite its low-dimensional character, the effective 1D system inherits the topological properties of higher-dimensional h-HOTI, including its topological invariant. Therefore, the projected corner states, localized either in the bulk or on the edge of the 1D array, are induced and protected by the symmetries of the original 4D system. We can therefore conclude that the dimensional reduction can be used to engineer new kinds of topological phases characterized by the nonlocal topological invariants, which ensure the presence of topological states protected by the nonlocal symmetries, which are ensured by long-range correlations of parameters within the array.

## Experimental Realization

As the next step we performed experimental testing of our theoretical predictions and designed a 1D DAA supporting localized topological zero-energy states. The dimensional reduction allows for one more simplification of significant relevance for the experimental realization of D-dimensional HOTIs, whose number of sites increases rapidly as ${\left(2N\right)}^{D}$, where N is the number of unit cells along one edge of the finite (hyper-) cubic array: The size of the system can be dramatically reduced by cutting the array at any of the weak bonds ($\tau_{n}\approx 0$) in the projected 1D DAA lattice without affecting the topological states due to their strong localization, provided that the truncation is far enough from their localization site. The simulated results for the truncated 1D lattice with only 20 sites (out of 1,296 original sites in 4D) are shown in Fig. 2 *B* and *C*, and its spectrum is seen to retain a gapped character with two fourth-order corner states pinned to zero energy (anchored-site corner mode slightly moves away from zero energy due to the intentional weak on-site perturbation). Note that we intentionally cut the chain after 20th site to demonstrate stability of the corner modes against cutting not at exactly vanishing bond (e.g., 30th site). Inspection of the probability distribution ${\left|\psi \right|}^{2}$ (Fig. 2*C*) confirms that one of the “zero-energy” states is localized at the boundary (the anchor site) and the other one is localized in the bulk at the 10th site.

**Fig. 3.**
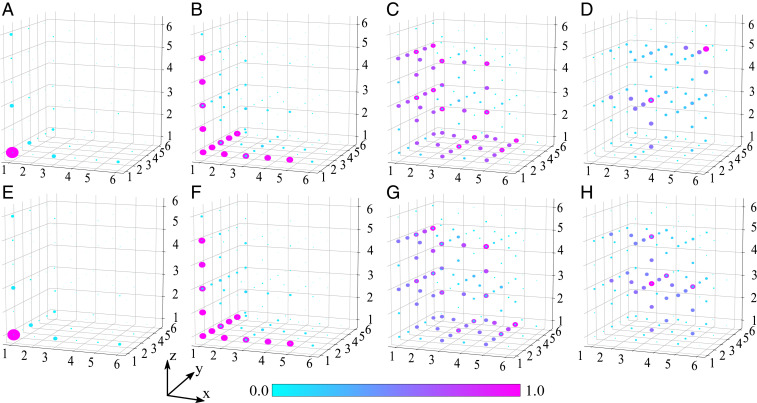
The effect of truncation of effective 1D lattice. (*A*–*D*) The intensity distributions for four different eigenstates of the 1D array of 30 sites, indicated by numbers 1 to 4 in Fig. 2*B*, projected back to 4D by the inverse Lanczos transformation. The modes clearly represent corner states, as well as reminiscent of hinge, surface, and hypersurface states in 4D, respectively. (*E*–*H*) The same, but for the case of 1D array truncated to 20 sites. The projections on the xyz hyperplane only are shown.

To confirm that the topological modes of 1D DAA are not affected by the truncation, we performed an inverse Lanczos transformation and mapped the eigenstates of the truncated DAA back to 4D. To this aim, we assigned zero intensities to the missing sites of the truncated 1D lattice to restore the number of degrees of freedom. The comparison of the field distribution on the *xyz* hyperplane for the boundary modes of different order (indicated by numbers in Fig. 2*B*) is shown in Fig. 3, where the results for the lattice truncated to 20 sites (Fig. 3 *E*–*H*) is plotted alongside selected (geometrically closes) eigenmodes of the exact (nontruncated) model (Fig. 3 *A*–*D*). The comparison reveals no difference in the field distributions not only for 4D corner states but even for selected lower-order hinge and surface states, and only the hypersurface states are clearly affected. Thus, the modes, in general, recover their field profiles in the projected DAA system, and they still appear separated by the reminiscent of the bandgaps indicated by colored regions in Fig. 2*B*. This further proves the stability of the localized topological modes in the DAA to truncations, which makes fabrication of systems supporting such designer topological states practically feasible even for the original higher-dimensional systems with very large number of degrees of freedom.

Clearly, after the truncation of the array due to reduction in the number of degrees of freedom only some of the modes are retained. Since some of these states have an extended distribution in the 1D array, it is not surprising that their spectrum is slightly perturbed (due to weak coupling with the rest of the array). The rest of the states, which are primarily localized in the removed part of the lattice, are lost during the cutting procedure (see *SI Appendix*, section S7 for more detailed discussion).

**Fig. 4.**
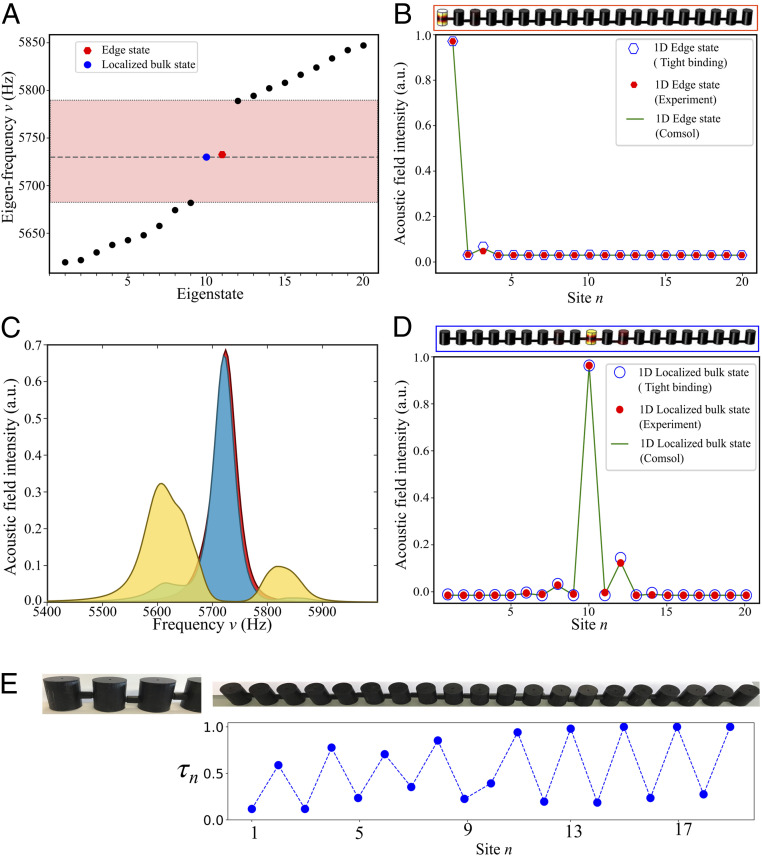
Band spectra and “zero-energy” modes of the truncated 1D DAA of acoustic resonators mapped from 4D h-HOTI. (*A*) Band spectra of the effective 1D model truncated to 20 sites obtained using first-principle FEM calculations. (*B*) Intensity distributions corresponding to the projected fourth-order topological corner mode localized at the edge of the array, as found from first-principle simulations (green line), experimental measurements (red dots), and tight-binding calculations (blue circles), respectively. (*C*) Experimentally measured density of states in the array. Yellow, blue, and red regions are color-coded to represent extended bulk, localized bulk, and localized edge modes. (*D*) Intensity distributions corresponding to the projected fourth-order topological corner mode localized in the bulk of the 1D array as found from first-principle simulations (green line), experimental measurements (red dots), and tight-binding calculations (blue circles), respectively. (*E*) (*Upper*) The 1D array fabricated using 3D printing. (*Lower*) The hopping amplitudes $\tau_{n}$ as a function of site position. The amplitudes are normalized to the maximal hopping amplitude 102.3Hz, and the onsite energy of the array is $\omega_{0}\approx 5,720\text{Hz}$.

To confirm the experimental feasibility of the dimensional reduction for the h-HOTI, we designed an acoustic analog of DAA of resonators emulating the 1D TBM using the finite-element method (FEM) software COMSOL Multiphysics (Acoustic Module). The individual resonators have the same height of 3.0 cm, so that the lowest-frequency resonance, corresponding to an odd pressure profile in the vertical (axial) direction (with a single node at the center), arises at f∼5,720 Hz. Coupling is introduced by adding narrow channel waveguides connecting the resonators in the array. The coupling strength between resonators (the hopping in the TBM) is modulated aperiodically in accordance with the mapping by vertically shifting the position of the connectors with respect to the node of the resonant mode. The simulation results for the system of 20 coupled resonators are presented in Fig. 4.

Fig. 4 *B* and *D* shows the acoustic field intensity of the topological modes localized at the anchor site and in the bulk, both stemming from the two corner states of the original 4D h-HOTI. Fig. 4*A* confirms that the spectral positions of these modes are indeed localized in the midgap (with a small deliberate shift for the edge state), while the extended bulk modes of the 1D array are gapped, which agrees with the results of the TBM in Fig. 2*B* (the experimental data on coupling strength can be found in *SI Appendix*, section S8).

The designed DAA of 20 coupled acoustic resonators was fabricated with the use of a high-resolution stereolithographic 3D printing (*Methods*). The resonators with connectors attached to them were printed and snapped together using interlocking features deliberately introduced in the design, thus allowing for the assembly of a rigid and stable array. The assembled structure of 20 resonators is shown in Fig. 4*E*. The modes were probed with a local excitation in each resonator by placing a speaker at holes introduced on the bottom of every resonator. The strength of the local response was measured using a directional microphone attached to the second hole on the top of the resonators. The holes were small enough not to introduce excessive loss, but sufficiently large to probe the acoustic pressure field inside the resonators.

The frequency-response spectra for selected groups of resonators - the anchor boundary site (red band), the 10th resonator in the bulk (blue band), and the average over all other bulk sites (yellow bands), are shown in Fig. 4*C* by color-coded bands, and clearly reveal two distinct types of states (see *Methods* for detailed measurement and data analysis). The one type is represented by two midgap states localized to the boundary and in the bulk at the 10th resonator, respectively. These modes correspond to the topological zero-energy states protected by nonlocal symmetries in the DAA. Direct measurement of the field profiles corresponding to the excitation at the terminal anchor site with the frequency close to the single-resonator (“zero-energy”) frequency confirms that the state is highly localized at the boundary of the array (Fig. 4*B*). Similarly, the second 4D corner state was directly excited by driving the 10th resonator at the “zero-energy” frequency, and it was found to strongly localize in the bulk near the respective site (Fig. 4*D*).

The other type of states (yellow bands in Fig. 4*C*) represents delocalized states of the 1D array, which are spectrally gapped and corresponding to hinge states of the 4D system adjacent to the anchor site. Despite the finite bandwidth of the resonances, of about 50 Hz, primarily due to losses induced by the leakage of sound through the probe holes and absorption in the resin, the localized topological states clearly retain their properties, i.e., a strong localization in the array and well-defined spectral position centered at “zero energy.” The resultant experimentally measured quality factors of the individual resonators are within the range of 50 to 60, and therefore the corresponding finite lifetimes of the modes of the structure are long enough not to alter their topological nature, making them clearly observable in our 1D array (see the discussion about the quality factor in *SI Appendix*, section S9).

## Discussion

In this work we have introduced a class of topological systems, DAAs, which support localized topological states protected by nonlocal symmetries and whose topology is inherited from higher dimensions. As an example, we considered the 1D DAA emulating 4D HOTI with quantized hexadecapolar multipole moment. We have theoretically mapped the 4D h-HOTI system onto DAA described by 1D TBM with the use of Lanczos tridiagonalization, which was then experimentally realized in an array of coupled acoustic resonators. We have proven that higher-dimensional topological corner states arise in the resultant projected 1D DAA in the form of localized resonant modes confined to the edge of the system and in the bulk. More importantly, we have shown that these modes retain their spectral features protected by chiral symmetry and reflection symmetries, which assume a nonlocal character in the DAA and establish a correlation of parameters within the array. Thus, the symmetries inherited from the original 4D system are mapped into 1D and ensure the very existence and spectral stability of the localized topological modes in 1D DAA.

The possibility to engineer multiple localized resonances via dimensional reduction and their unique features, such as precise spectral properties and topological robustness, open remarkable opportunities for practical applications, from robust resonators to sensors and aperiodic topological lasers. Even wider opportunities can be uncovered once similar mapping procedures are developed to produce 2D and 3D DAAs, which can provide more degrees of freedom to manipulate projected topological states propagating along edges and hinges of such the systems. More broadly, our work shows how higher-dimension topological physics may be mapped into easily realizable devices using dimensionality reduction, opening unique opportunities to implement and verify the exotic features of higher-dimension topological physics in quantum and classical systems. More importantly, our work reveals a class of topological systems where nonlocal symmetries give rise to nonvanishing topological invariants inherited from higher dimensions and having a completely different meaning with similar implications.

## Methods

### Structure Design, 3D Printing, and Generic Measurements.

The unit cell designs of the topological expanded lattice are shown in Fig. 4*E* with lattice constant $a_{0}$ = 33 mm, height $H_{0}$ = 30.00 mm, and radius $r_{0}$ = 10 mm. The connectors between the cylinders are radial channels with diameter $d_{\gamma }$ = 5 mm. The unit cells and boundary cells were fabricated using the B9Creator v1.2 3D printer. All cells were made with acrylic-based light-activated resin, a type of plastic that hardens when exposed to ultraviolet light. Each cell was printed with a sufficient thickness to ensure a hard wall boundary condition and narrow probe channels were intentionally introduced on the top and bottom of each cylinder to excite and measure local pressure intensity at each site. The diameter of each port is 2.50 mm with a height of 2.20 mm. When not in use, the probe channels were sealed with plumber’s putty. Each unit cell and boundary cell were printed one at a time and the models were designed specifically to interlock tightly with each other. The expanded structure shown in Fig. 4*E* contains 20 unit cells. For all measurements, a frequency generator and fast Fourier transform spectrum analyzer scripted in LabVIEW were used.

### Numerical Method.

Finite element solver COMSOL Multiphysics 5.2a with the Acoustic Module was used to perform full-wave simulation. In the acoustic propagation wave equation, the speed of sound was set as 343.2 m/s and density of air as 1.225 kg/m^3^. Other dimensional parameters of the structure are the same as the fabricated parameters.

## Supplementary Material

Supplementary File

## Data Availability

All study data are included in the article and/or *SI Appendix*.
