# Supplementary material for: Nonlocal topological insulators: Deterministic aperiodic arrays supporting localized topological states protected by nonlocal symmetries
Source: Proc Natl Acad Sci U S A. 2021 Aug 19;118(34):e2100691118. doi: 10.1073/pnas.2100691118 (PMC8403875; doi:10.1073/pnas.2100691118)
Supplement: Supplementary File [file pnas.2100691118.sapp.pdf]

Supplementary material for

Nonlocal Topological Insulators: Deterministic Aperiodic Arrays Supporting Localized Topological States Protected by Nonlocal Symmetries

Kai Chen[1,2], Matthew Weiner[1,2], Mengyao Li[1,2], Xiang Ni[3], Andrea Alù[3,2,1], and Alexander B. Khanikaev[1,2,*]

[1]Department of Electrical Engineering, Grove School of Engineering, City College of the City University of New York, 140th Street and Convent Avenue, New York, NY 10031, USA.

[2]Physics Program, Graduate Center of the City University of New York, New York, NY 10016, USA.

[3]Photonics Initiative, Advanced Science Research Center, City University of New York, New York, NY 10031, USA

*Alexander B. Khanikaev

Email: akhanikaev@ccny.cuny.edu

S1. Hamiltonian and symmetries

The specific form of four-dimensional momentum-space Hamiltonian of hexadecapole topological insulator (HTI) is gauge dependent and can be chosen as

$$\hat{H}(\boldsymbol{k}) = \sum_n \lambda_n \sin(k_n)\, \Gamma_{2n-1}^{\prime\prime} + [\gamma_n + \lambda_n \cos(k_n)]\Gamma_{2n}^{\prime\prime} \quad, n = 1,2,3,4, \qquad (1)$$

where λ_n and γ_n are nearest neighbor inter-cell hopping term and intra-cell hopping term in the n direction, and assume lattice constant is one. $\Gamma_i^{\prime\prime} = \sigma_3 \otimes \Gamma_i^{\prime}$ for $i = 0,1,..,6$, $\Gamma_7^{\prime\prime} = \sigma_2 \otimes I_{8\times 8}$, $\Gamma_8^{\prime\prime} = \sigma_1 \otimes I_{8\times 8}$, $\Gamma_i^{\prime} = \sigma_3 \otimes \Gamma_i$ for $i = 0,1,2,3,4$, $\Gamma_5^{\prime} = \sigma_2 \otimes I_{4\times 4}$, $\Gamma_6^{\prime} = \sigma_1 \otimes I_{4\times 4}$ and $\Gamma_0 = \tau_3 \otimes \tau_0$, $\Gamma_i = -\tau_2 \otimes \tau_i, i = 1,2,3$, $\Gamma_4 = \tau_1 \otimes \tau_0$, where σ_is and τ_is are the Pauli matrices. Accordingly, the eigenvalues of the Hamiltonian are

$$E(\boldsymbol{k}) = \pm\sqrt{\sum_n \lambda_n^2 + \gamma_n^2 + 2\gamma_n \lambda_n \cos(k_n)}. \qquad (2)$$

The energy bands of HTI unit cell are obtained in Fig. S1 (a), and the according Brillouin zone projected in a specific 3D space is sketched in Fig. S1(b). The Hamiltonian in Eq. (1) has reflection symmetries in all directions up to gauge transformation(1). The matrix representations of reflection symmetries under the chosen gauge are expressed as

$$\hat{M}_1 = \sigma_0 \otimes \sigma_0 \otimes \tau_1 \otimes \tau_3,$$
$$\hat{M}_2 = \sigma_0 \otimes \sigma_0 \otimes \tau_1 \otimes \tau_1,$$
$$\hat{M}_3 = \sigma_0 \otimes \sigma_1 \otimes \tau_3 \otimes \tau_0,$$
$$\hat{M}_4 = \sigma_1 \otimes \sigma_3 \otimes \tau_3 \otimes \tau_0. \qquad (3)$$

These symmetry operators meet the anti-commuting relation because of the synthetic magnetic flux π on each plaquette of the crystal,

$\{\widehat{M}_n, \widehat{M}_m\} = 0, n, m = 1,2,3,4\ n \neq m.$ (4)

The operator of inversion symmetry for a spinless HTI is constructed by

$\hat{I} = \widehat{M}_k \widehat{M}_l \widehat{M}_m \widehat{M}_n,$ (5)

where $k, l, m, n = 1,2,3,4, k \neq l \neq m \neq n$. Because of anti-commuting reflection symmetries, $\hat{I}^2 = -1$, which is crucial to guarantee the degenerate energy bands. Furthermore, chiral symmetry ($\Gamma_0^{'''} \widehat{H} \Gamma_0^{'''-1} = -\widehat{H}$) makes sure the energy spectrum is always symmetric along zero energy $\epsilon = 0$ (Fig. S1(a)).

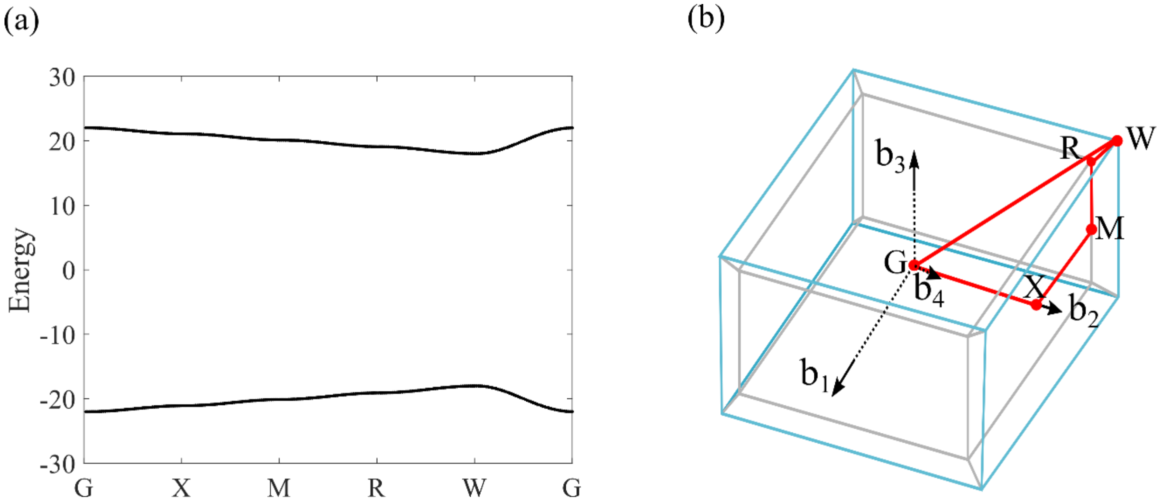

Fig. S1 (a) Energy bands of 4D hexadecapolar HOTI along high symmetric points in 4D Brillouin zone. (b) Schlegel diagram of 4D Brillouin zone of hypercubic lattice.

S2. Higher-order Wannier bands

Wilson loop eigenvalue problem for HTI can be written as

$$W_{2\pi+p_n\leftarrow p_n,\boldsymbol{p}} |v_{n,\boldsymbol{p}}^i\rangle = e^{i2\pi v_{n,\boldsymbol{p}}^i} |v_{n,\boldsymbol{p}}^i\rangle, \quad (6)$$

where$W_{2\pi+p_n\leftarrow p_n} = G(2\pi + p_n, 2\pi + p_n - \delta p_n) \ldots G(p_n + 2\delta p_n, p_n + \delta p_n) G(p_n + \delta p_n, p_n)$,
and $G_{i,i'}(p_n + \delta p_n, p_n) = \sum_\alpha u_{p_n+\delta p_n,\alpha}^{i}{}^* u_{p_n,\alpha}^{i'}$, $i, i' = 1,2, \ldots, N_o, \alpha = 1,2, \ldots, 16, N_o$ is the maximum number of occupied energy bands (largest-frequency bands of interest in acoustic metamaterial). $\boldsymbol{p} = (p_k, p_l, p_m, p_n), p_k, p_l, p_m, p_n = 0, \delta p, \ldots, (N_p - 1)\delta p$, each reciprocal direction of first Brillouin zone is discretized by N_p steps, and $\delta p = 2\pi/N_p$. $|v_{n,\boldsymbol{p}}^i\rangle$ is the Wannier state, and $v_{n,\boldsymbol{p}}^i$ is the Wannier center of the ith occupied band with the Bloch eigenstate $|u_{\boldsymbol{p},\alpha}^i\rangle$, which describes the average position of the particle from the center of the unit cell in the direction p_n at momentum vector $\boldsymbol{p}$. The polarization p_n at a particular hinge n is procured by integrating Wannier center over first Brillouin zone and summation over occupied bands, the alternative form in numerical calculation is,

$$p_n = \frac{1}{2\pi} \left(\frac{1}{N_p}\right)^3 \sum_{i,p_k,p_l,p_m} v_{n,\boldsymbol{p}}^i, \quad (7)$$

which is quantized as either 0 or 1/2 because of reflection symmetries. Similarly, polarizations in other directions can be also abstained based on the same procedures. Note to have a nonzero higher order topology in the system defined in the following text, the total polarization of a finite bulk is zero.

In higher-order topological insulator, the bulk states might not directly possess the topological phase characterized by the 1st order Wannier bands, instead they inherit the higher order topological quantity from that of lower dimensional states. To determine the topological phase of boundary state which is one dimensional lower than its host bulk, 2nd order Wilson loop (nested Wilson loop) has been invented recently(1, 2) which is performed over the subspace of Wannier-sector, for example, on Wannier-sector $\pm\nu_n$ with N_{w^1} 1st order Wannier bands considered, and along p_m in the Brillouin zone,

$$W_{m,\boldsymbol{p}}^{\pm n} = F_{\boldsymbol{p}}^{\pm n}(2\pi + p_m, 2\pi + p_m - \delta p_m) \dots F_{\boldsymbol{p}}^{\pm n}(p_m + 2\delta p_m, p_m + \delta p_m) F_{\boldsymbol{p}}^{\pm n}(p_m + \delta p_m, p_m), \quad (8)$$

in which $\left[F_{\boldsymbol{p}}^{\pm n}(p_m + \delta p_m, p_m)\right]^{j,j'} = \langle w_{\pm n, p_m + \delta p_m}^{j} | w_{\pm n, p_m}^{j'} \rangle,\ j, j' = 1,2, \dots, N_{w^1}$, where the 1st order Wannier state over Wannier-sector $\pm\nu_n$ is defined as

$$\left| w_{\pm n,\boldsymbol{p}}^{j} \right\rangle = \sum_{i}^{N_{occ}} \left| u_{\boldsymbol{p}}^{i} \right\rangle \left[\nu_{n,\boldsymbol{p}}^{\pm j} \right]^{i}, \quad (9)$$

where $\left[\nu_{n,\boldsymbol{p}}^{j}\right]^{i}$ is the ith component of the Wannier eigenstate $|\nu_{n,\boldsymbol{p}}^{j}\rangle$. Subsequently, we have the eigenvalue equation for the 2nd order Wilson loop

$$W_{m,\boldsymbol{p}}^{\pm n} |\nu_{m,\boldsymbol{p}}^{\pm n,j}\rangle = e^{i2\pi \nu_{m,\boldsymbol{p}}^{\pm n,j}} |\nu_{m,\boldsymbol{p}}^{\pm n,j}\rangle, \quad (10)$$

in which $\nu_{m,\boldsymbol{p}}^{\pm n,j}$ is the 2nd order Wannier center of jth (1st order) Wannier band in the Wannier sector $\pm\nu_n$. The polarization over the Wannier sector $\pm\nu_n$ is given by the equation,

$$p_m^{\pm n} = \frac{1}{2\pi} \left(\frac{1}{N_p} \right)^3 \sum_{j, p_n, p_k, p_l} \nu_{m,\boldsymbol{p}}^{\pm n,j}, \quad (11)$$

Note that index p_n in Eq. (11) is redundant since $\nu_{m,\boldsymbol{p}}^{\pm n,j}$ is independent of it, however, we keep this index to have a traceable and compacted form of polarization which can be easily extended to the case in higher dimension, as we show in the following text. The quadrupole moment of a specific surface nm, is defined as

$$q_{nm} = 2 p_n^{\pm m} p_m^{\pm n}, n, m = 1,2,3,4, n \neq m, \quad (12)$$

and it is quantized as either $1/2$ or 0 because of the constraint by reflection symmetries. Continuing the above process, to get the topological invariant for the boundary states two dimension lower compared to the bulk, 3rd order Wilson loop is constructed over the subspace of Wannier-sector $\pm\nu_m^{\pm n}$ with N_{w^2} 2nd order Wannier bands considered, which is defined along p_l in the Brillouin zone,

$$W_{l,\boldsymbol{p}}^{\pm m,\pm n} = F_{\boldsymbol{p}}^{\pm m,\pm n}(2\pi + p_l, 2\pi + p_l - \delta p_l) \dots F_{\boldsymbol{p}}^{\pm m,\pm n}(p_l + 2\delta p_l, p_l + \delta p_l) F_{\boldsymbol{p}}^{\pm m,\pm n}(p_l + \delta p_l, p_l), \quad (13)$$

in which $\left[F_{\boldsymbol{p}}^{\pm m,\pm n}(p_l + \delta p_l, p_l)\right]^{s,s'} = \langle w_{\pm m,\pm n, p_l + \delta p_l}^{s} | w_{\pm m,\pm n, p_l}^{s'} \rangle,\ s, s' = 1,2, \dots, N_{w^2}$, where the 2nd order Wannier state over Wannier-sector $\pm\nu_m^{\pm n}$ is defined as

$$\left| w_{\pm m,\pm \mathrm{n},\boldsymbol{p}}^{s} \right\rangle = \sum_{j}^{N_{w^1}} \left| w_{\pm n,\boldsymbol{p}}^{j} \right\rangle \left[\nu_{m,\boldsymbol{p}}^{\pm n,\pm s} \right]^{j}. \quad (14)$$

Note $\left[v_{m,\boldsymbol{p}}^{\pm n,s}\right]^j$ is the jth component of the 2nd order Wilson loop eigenstate $|v_{m,\boldsymbol{p}}^{\pm n,s}\rangle$. The eigenvalue equation for the 3rd order Wilson loop is expressed as

$$W_{l,\boldsymbol{p}}^{\pm m,\pm n}|v_{l,\boldsymbol{p}}^{\pm m,\pm n,s}\rangle = e^{i2\pi v_{l,\boldsymbol{p}}^{\pm m,\pm n,s}}|v_{l,\boldsymbol{p}}^{\pm m,\pm n,s}\rangle, \tag{15}$$

in which $v_{l,\boldsymbol{p}}^{\pm m,\pm n,s}$ is the 3rd order Wannier center of sth (2nd order) Wannier band in the category of Wannier-sector $\pm v_m^{\pm n}$. The polarization over the Wannier-sector $\pm v_m^{\pm n}$ is given by the equation,

$$p_l^{\pm m,\pm n} = \frac{1}{2\pi}\left(\frac{1}{N_p}\right)^3 \sum_{s,p_m,p_n,p_k} v_{l,\boldsymbol{p}}^{\pm m,\pm n,s}. \tag{16}$$

The octupole moment on an arbitrary surface lmn can be obtained by

$$o_{lmn} = 4p_l^{\pm m,\pm n} p_m^{\pm n,\pm l} p_n^{\pm l,\pm m}, l,m,n = 1,2,3,4, l \neq m \neq n, \tag{17}$$

which is quantized either to be 1/2 or 0 because of the conservation of reflection symmetries.

Finally, we extend the topological invariant for the boundary states three dimensional lower than their bulk by inventing the tool which is the 4th order Wilson loop over the subspace of Wannier-sector $\pm v_l^{\pm m,\pm n,}$ with N_{w^3} 3rd order Wannier bands considered, and along p_k in the Brillouin zone,

$$W_{k,\boldsymbol{p}}^{\pm l,\pm m,\pm n} = F_{\boldsymbol{p}}^{\pm l,\pm m,\pm n}(2\pi + p_k, 2\pi + p_k - \delta p_k) \dots F_{\boldsymbol{p}}^{\pm l,\pm m,\pm n}(p_k + 2\delta p_k, p_k + \delta p_k) F_{\boldsymbol{p}}^{\pm l,\pm m,\pm n}(p_k + \delta p_k, p_k), \tag{18}$$

in which $\left[F_{\boldsymbol{p}}^{\pm l,\pm m,\pm m}(p_k + \delta p_k, p_k)\right]^{t,t'} = \langle w_{\pm l,\pm m,\pm n,p_k+\delta p_k}^t | w_{\pm l,\pm m,\pm n,p_k}^{t'}\rangle$, $t,t' = 1,2,\dots,N_{w^3}$, where the 3rd order Wannier state over Wannier-sector $\pm v_l^{\pm m,\pm n}$ is defined as

$$\left|w_{\pm l,\pm \mathrm{m},\pm \mathrm{n},\boldsymbol{p}}^t\right\rangle = \sum_s^{N_{w^2}} \left|w_{\pm m,\pm n,\boldsymbol{p}}^s\right\rangle \left[v_{l,\boldsymbol{p}}^{\pm m,\pm n,\pm t}\right]^s. \tag{19}$$

Note $\left[v_{l,\boldsymbol{p}}^{\pm m,\pm n,t}\right]^s$ is the sth component of the 3rd order Wilson loop eigenstate$|v_{l,\boldsymbol{p}}^{\pm m,\pm n,t}\rangle$. Finally, we have the eigenvalue equation for the 4th order Wilson loop

$$W_{k,\boldsymbol{p}}^{\pm l,\pm m,\pm n}|v_{k,\boldsymbol{p}}^{\pm l,\pm m,\pm n,t}\rangle = e^{i2\pi v_{k,\boldsymbol{p}}^{\pm l,\pm m,\pm n,t}}|v_{k,\boldsymbol{p}}^{\pm l,\pm m,\pm n,t}\rangle, \tag{20}$$

in which $v_{k,\boldsymbol{p}}^{\pm l,\pm m,\pm n,t}$ is the 4th order Wannier center of tth (3rd order) Wannier band in the category of Wannier-sector $\pm v_l^{\pm m,\pm n}$. The polarization over the Wannier-sector $\pm v_l^{\pm m,\pm n}$ is given by the equation,

$$p_k^{\pm l,\pm m,\pm n} = \frac{1}{2\pi}\left(\frac{1}{N_p}\right)^3 \sum_{t,p_l,p_m,p_n} v_{k,\boldsymbol{p}}^{\pm l,\pm m,\pm n,t}, \tag{21}$$

Due to the constraint of reflection symmetries, the hexadecapole moment of a 4D bulk is defined as

$$h_{klmn} = 8p_k^{\pm l,\pm m,\pm n} p_l^{\pm m\pm n,\pm k} p_m^{\pm n,\pm k,\pm l} p_n^{\pm k,\pm k,\pm m}, k,l,m,n = 1,2,3,4, k \neq l \neq m \neq n$$
(22)

and it is quantized as either $1/2$ or 0. The topological evidences of nontrivial quantized hexadecapole moment shown in Fig. S2(d) are the fractional corner charges localized at the 16 corners of the hypercubic lattice, as demonstrated by both theory and experiment in the manuscript.

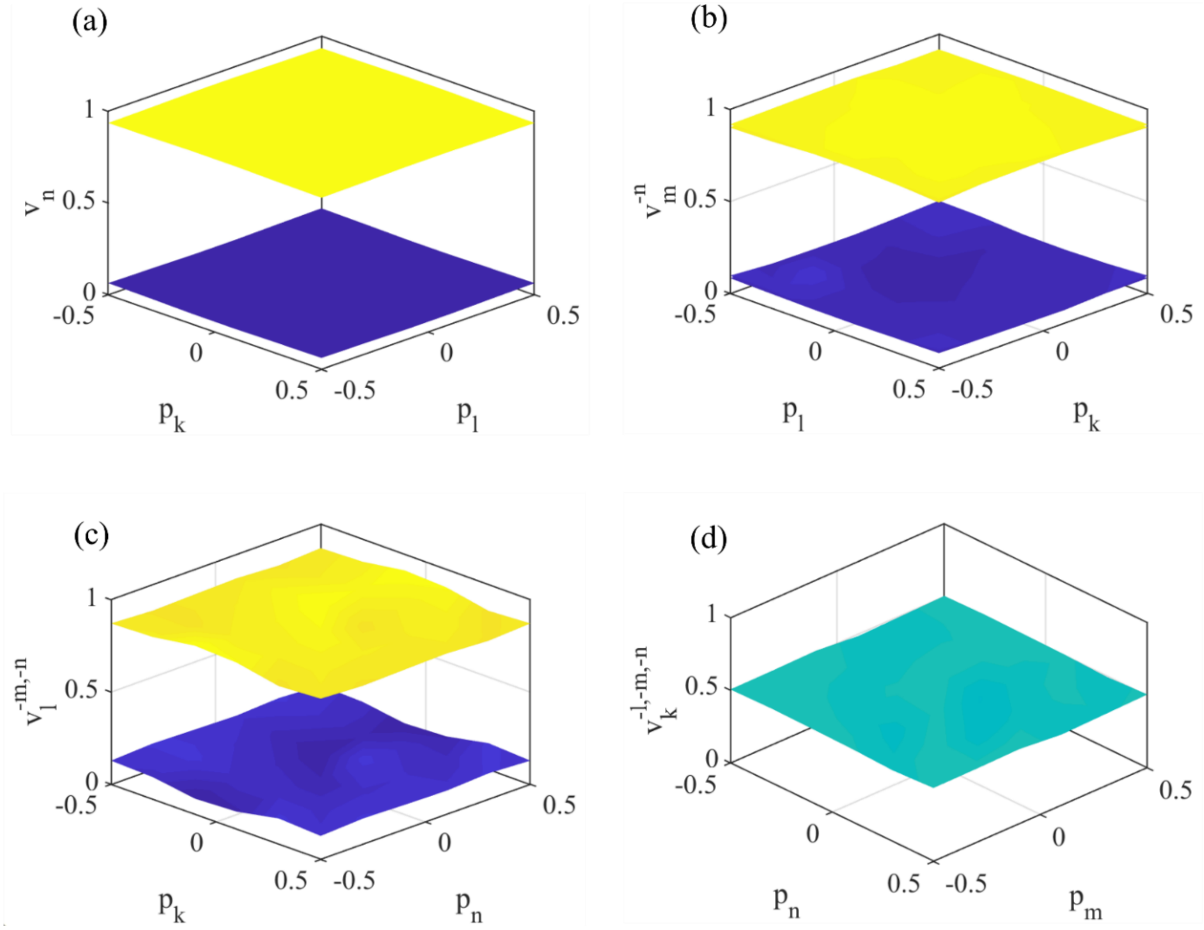

Fig. S2. Multiple order topology of nontrivial HTI based on Hamiltonian in Eq. (1). (a) First order Wannier bands, (b) second order Wannier bands and (c) third order Wannier bands which are gapped everywhere in Brillouin zone, these spectra have degenerate bands because of anti-commuting reflection symmetries, and the gapped properties of the spectra are enforced by inversion symmetry. (d) Fourth order Wannier band which is quantized as 1/2 everywhere. The parameters adopted in TBM calculation are $\lambda_n = 10, \gamma_n = 1, n = 1,2,3,4$, infinitesimal disorder is introduced to facility the numerical calculation of Wannier bands.

S3: Bulk localization of corner modes

The degree of localization can be quantified by defining the inverse participation ratio (IPR) of eigenstates(3), i.e., $IPR(|\varphi >) := \sum_{n=1}^{N_{sites}} |\langle n|\varphi\rangle|^4$.

Take { $|\varphi_i\rangle$ for i = 1, 2…N_{corner} } as corner mode eigenstates of the 4-dimensional Hamiltonian H_{4d} and { $|m\rangle$ for m = 1, 2…N_{sites} } as basis of the Hamiltonian, then $H_{4d} = \sum_{m,n=1}^{N_{sites}} H_{mn}|m >< n|$. Using Lanczos transformation, we get a series of new basis$|\widetilde{m} > = U|m >$, then it's easy to get the relation $|\tilde{\varphi}_i > = U|\varphi_i\rangle$ and $H_{1d} = \sum_{m,n=1}^{N_{sites}} h_{mn}|\widetilde{m} >< \tilde{n}|$, where $h_{mn} = (UHU^+)_{mn}$.

$$IPR(|\tilde{\varphi}_i >) = \sum_{n=1}^{N_{sites}} |\langle\tilde{n}|\tilde{\varphi}_i\rangle|^4 = \sum_{n=1}^{N_{sites}} |\langle n|UU^+|\varphi_i\rangle|^4 = \sum_{n=1}^{N_{sites}} |\langle n|\varphi_i\rangle|^4 = IPR(|\varphi_i >)$$

If { $|\varphi_i >$ } are localized for 4d lattice then {$|\tilde{\varphi}_i >$} are localized for 1d lattice.

Since we introduced a tiny perturbation $\delta = 0.2t_1$ in (1) to shift one corner mode ($|\varphi_1 >$) from zero energy by δ in the 4d lattice model to guarantee this corner mode is orthogonal with other corner modes, in such way the corresponding projected state, according to the proof above, will be the edge mode ($|\tilde{\varphi}_1 >$) of 1d system.

$\langle\varphi_1|\varphi_i\rangle = 0$ for i = 2, 3 …16, after the Lanczos Transformation we have $\langle\tilde{\varphi}_1|\tilde{\varphi}_i\rangle = 0$ for i = 2, 3 …16, which means other zero energy modes will either localize at bulk of 1d system far away from the sites where $|\tilde{\varphi}_1 >$ is dominant, or at least orthogonal with $|\tilde{\varphi}_1 >$.

S4: Amplitude Distribution

The Lanczos transformation maps four-dimensional (4d) localized modes to localized modes of the one-dimensional (1d) system and 4d delocalized modes to 1d delocalized modes. We plot 16 localized eigenstates of the 1d system in Fig.S4, these states are mapped from the 4d corner modes. There is one eigenstate localized at the edge while the 15 other eigenstates are localized in the bulk.

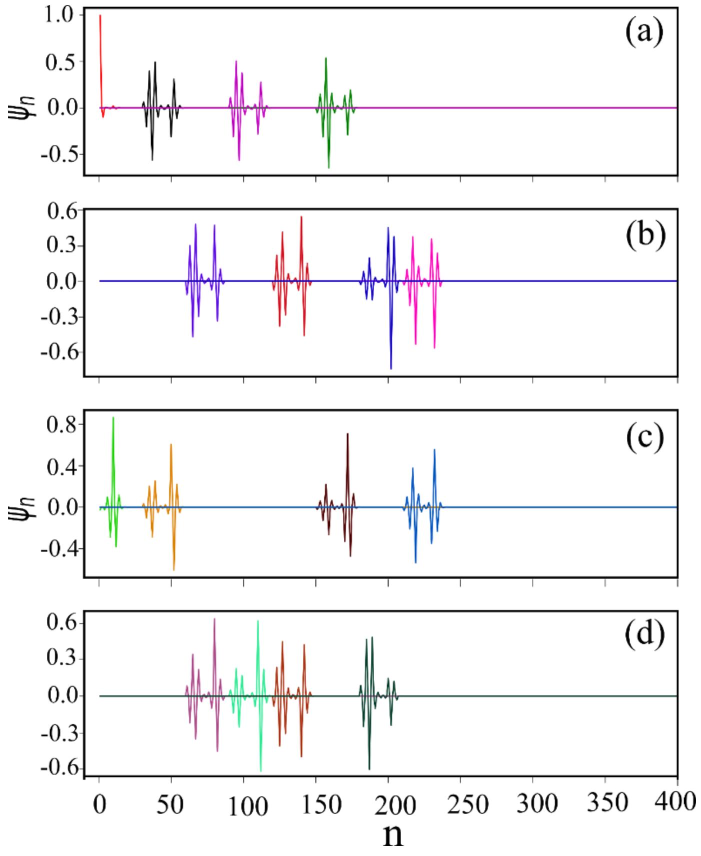

Fig. S3: Amplitude distribution of 4d corner modes in 1d lattice. $L_x = L_y = L_z = L_w = 3$, $t_1 = 1.0$, $t_2=10$. Red line in (a) correspond to localized edge mode in the main text, green line in (c) correspond localized bulk mode in the main text.

S5: Lanczos Tridiagonalization of Chiral Symmetry and Reflection Symmetries

The emergence of a quantized multipole moment and corner states is deeply related to the symmetries of the system, i.e., the presence of anti-commuting reflection symmetries in the 4D h-HOTI. Similarly, the "zero-energy" of the states is ensured by chiral symmetry, which stems from the fact that in the 4D h-HOTI the sites that belong to the same sublattice do not couple with each other. In addition, the Hamiltonian (1) possesses reflection symmetries that anti-commutate with each other because of the flux of π in each plaquette of the hypercubic lattice. The chiral symmetry of the system, on the other hand, with matrix representation $\hat{\Gamma} = \sigma_3 \otimes \sigma_3 \otimes \sigma_3 \otimes \sigma_0$, is expressed as $\hat{\Gamma}\hat{\mathcal{H}}_{4\mathrm{D}}\,\hat{\Gamma}^{-1} = -\hat{\mathcal{H}}_{4D}$, which ensures the overall symmetry of the spectrum and "zero-energy" of the corner states. The fact that this property is retained during the dimensional reduction implies that the chiral symmetries is preserved in new form and can be expressed as $\hat{U}_L\hat{\Gamma}\hat{U}_L^{-1}$. Indeed, due to its local character, the chiral symmetry can be written as a symmetry operator for the finite h-HOTI as $\hat{\Gamma}^{finite} = I_{N\times N} \otimes \hat{\Gamma}$, where $I_{N\times N}$ is the N by N identity matrix and N is the number of degrees of freedom (unit cells), and thus is (block)-diagonal. The Lanczos transformation changes the local character of the chiral symmetry by mixing different sites (except the anchor site) $\hat{\Gamma}_{1D}^{eff} = \hat{U}_L\hat{\Gamma}^{finite}\hat{U}_L^{-1}$, thus inducing the new non-local form of chiral symmetry $\hat{\Gamma}_{1D}^{eff}\hat{\mathcal{H}}_{1D}^{eff}\,\hat{\Gamma}_{1D}^{eff^{-1}} = -\hat{\mathcal{H}}_{1D}^{eff}$ of the effective 1D Hamiltonian. This effective chiral symmetry of the 1D Hamiltonian plays the same role as the original chiral symmetry for 4D h-HOTI and it ensures spectral stability of the modes of

aperiodic 1D array. However, the non-local character of the symmetry operator $\hat{\Gamma}_{1D}^{eff}$ in 1D is reflected in the presence of correlations of parameters in different part of array, hopping amplitudes and on-site energies, within the 1D system, responsible for the "zero-energy" of the localized states. The reflection symmetries and the resultant quantized multipole moment can be similarly analyzed in the dimensionally reduced system, and the quantized multipole moment of the bulk bands in the finite array can be extracted from the respective wave functions. Thus, despite its low-dimensional character, the effective 1D system inherits the properties of higher-dimensional h-HOTI. Therefore, the projected corner states, localized either in the bulk or on the edge of the 1D array, are induced and protected by the symmetries of the original 4D system, thus ensuring their very existence and stability specific to topological systems.

The finite 4D lattice model satisfy chiral symmetry $\hat{\Gamma}^{finite}$,

$$\hat{\Gamma}^{finite}\hat{\mathcal{H}}_{4D}^{finite}{\hat{\Gamma}^{finite}}^{-1} = -\hat{\mathcal{H}}_{4D}^{finite},$$

Under Lanczos Tridiagonalization(LTD), $\hat{U}_L\hat{\mathcal{H}}_{4D}^{finite}\hat{U}_L^{-1} = \hat{\mathcal{H}}_{1D}^{eff}$, $\hat{U}_L\hat{\Gamma}^{finite}\hat{U}_L^{-1} = \hat{\Gamma}_{1D}^{eff}$

The chiral symmetry in the finite 4D lattice model is local operator. However, The LTD transformation maps the local operator to non-local operator (as shown in Fig.S4) and the effective 1D lattice Hamiltonian satisfies the same anti-commutation relation with the non-local operator, $\hat{\Gamma}_{1D}^{eff}\hat{\mathcal{H}}_{1D}^{eff}{\hat{\Gamma}_{1D}^{eff}}^{-1} = -\hat{\mathcal{H}}_{1D}^{eff}$. For the 4D lattice with 3^4 sites, the reflection symmetry $\hat{M}$,

$\hat{M} \in \{\hat{M}_x := I_{3X3} \otimes I_{3X3} \otimes I_{3X3} \otimes D \otimes \sigma_0 \otimes \sigma_0 \otimes \sigma_0 \otimes \sigma_1, \hat{M}_y := I_{3X3} \otimes I_{3X3} \otimes D \otimes I_{3X3} \otimes \sigma_0 \otimes \sigma_0 \otimes \sigma_2 \otimes \sigma_2, \hat{M}_z := I_{3X3} \otimes D \otimes I_{3X3} \otimes I_{3X3} \otimes \sigma_0 \otimes \sigma_1 \otimes \sigma_0 \otimes \sigma_3,\ \hat{M}_w := D \otimes I_{3X3} \otimes I_{3X3} \otimes I_{3X3} \otimes \sigma_1 \otimes \sigma_3 \otimes \sigma_0 \otimes \sigma_3 \}$,

Where $D = \begin{bmatrix} 0 & 0 & 1 \\ 0 & 1 & 0 \\ 1 & 0 & 0 \end{bmatrix}$ and the resultant quantized multipole moment, can be similarly analyzed in the dimensionally reduces system, and the quantized multipole moment of the bulk bands in the finite array can be extracted from the respective wave functions. Thus, despite its low-dimensional character, the effective 1D system indeed inherits all the characteristics of higher-dimensional h-HOTI. Therefore, the projected corner states, localized either in the bulk or on the edge of the 1D array, are induced and protected by the symmetries of the original 4D system, thus ensuring their very existence and stability that are specific to topological systems.

$$\hat{M}\hat{\mathcal{H}}_{4D}^{finite}\hat{M}^{-1} = \hat{\mathcal{H}}_{4D}^{finite},$$

LTD map reflection symmetry $\hat{M}$ to $\hat{U}_L\hat{M}\hat{U}_L^{-1} = \hat{M}^{1D}$, the reflection symmetry loss its locality to be a global operator as shown in Fig.S5.

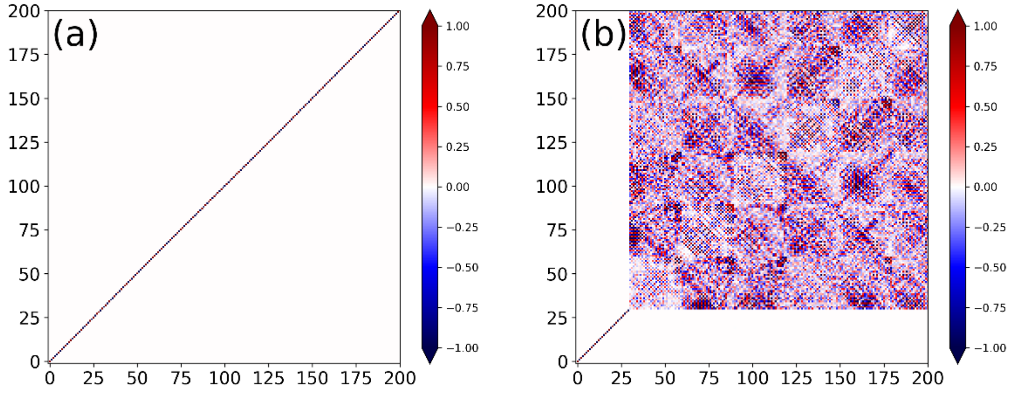

Fig. S4. Chiral symmetry under Lanczos Tridiagonalization. (a) Chiral symmetry matrix of the 4D lattice**. (b)** Chiral symmetry matrix of the 4D lattice after Lanzcos transformation. We only show a part of the chiral symmetry in order to show the matrix clearly.

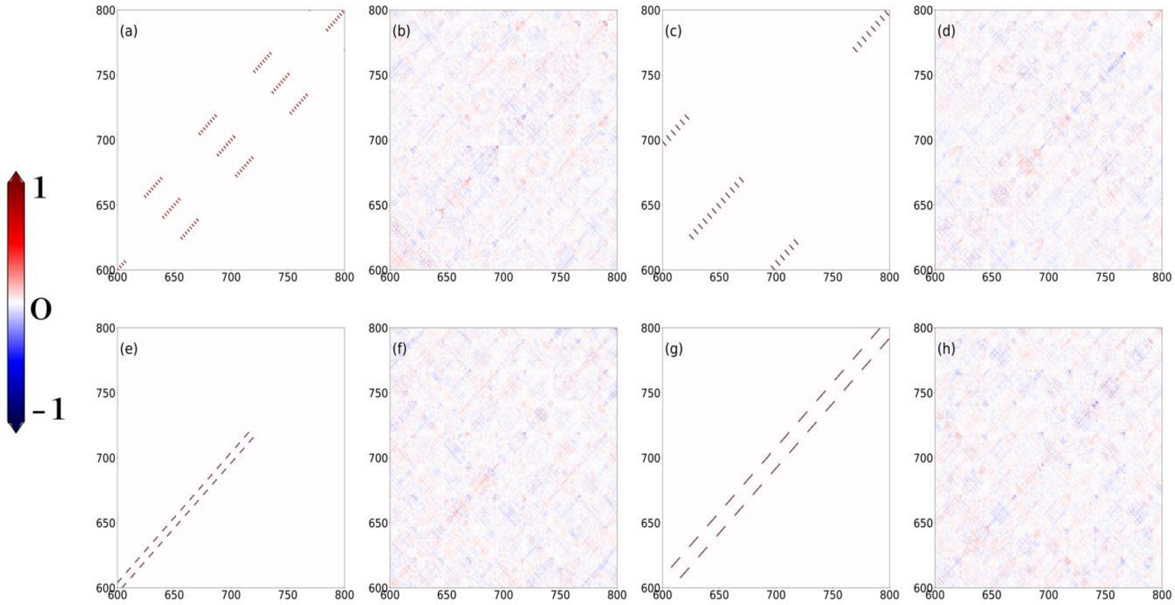

Fig. S5. Reflection symmetry under Lanczos Tridiagonalization. Lanzcos transformation (LTD) of reflection symmetry matrix of the 4D lattice. (a,b) LTD of $\widehat{M}_x$. (c,d) LTD of $\widehat{M}_y$. (e,f) LTD of $\widehat{M}_z$ and (g,h) LTD of $\widehat{M}_w$. We only show a part of the reflection symmetry in order to show the matrix clearly.

S6: Topological invariant (h_{DAA}) of deterministic aperiodic 1D array.

We use the real space formula of hexadecapole moment(4)

$$h_{xyzw} = \left(\frac{1}{2\pi} Im\, Tr\left[\ln \hat{P}^{+}\hat{D}\hat{P}\right] - \frac{n_f}{N}\, Tr[E]\right) modulo\ 1 \qquad (23)$$

to distinguish different phases for the 4D h-HOTI. The matrix $\hat{P} = \{|\alpha_1>, |\alpha_2>, \ldots, |\alpha_{N_{occ}}>\}$, where N_{occ} indicates the number of occupied (or negative energy for half-filling) states, and $|\alpha_n>$ are eigenstates of the finite 4D h-HOTI system with eigenenergies E_n .

$E = diag\{f(\overrightarrow{r_1}), f(\overrightarrow{r_2}), \ldots f(\overrightarrow{r_N})\}$, $\hat{D} = diag\{\exp\left[i2\pi\frac{f(\boldsymbol{r}_1)}{N}\right], \exp\left[i2\pi\frac{f(\boldsymbol{r}_2)}{N}\right], \ldots \exp\left[i2\pi\frac{f(\boldsymbol{r}_N)}{N}\right]\}$, $f(\boldsymbol{r}_k) = x_k y_k z_k w_k$ and $n_f = \frac{1}{2}$ is the filling in the system. The Lanczos transformation ($\hat{U}_L$) maps h_{xyzw} to be

$$h_{DAA} = \left(\frac{1}{2\pi} Im\, Tr\left[\ln \tilde{P}^{+}\tilde{D}\tilde{P}\right] - \frac{n_f}{N}\, Tr[E]\right) modulo\ 1 \qquad (24)$$

where $\tilde{P} = \{|\widetilde{\alpha_1}>, |\widetilde{\alpha_2}>, \ldots |\widetilde{\alpha_{N_{occc}}}>\}$ and $\tilde{D} = \hat{U}_L \hat{D} \hat{U}_L^{+}$. We argue the h_{xyzw} is a reasonable quantity to capture the topological transition of the 1D array since it depends only on the occupied eigenstates ($|\tilde{\alpha}>$) of the effective 1D array. We show h_{xyzw} and h_{DAA} in Fig. S6, two quantities have the same topological transition region, which means the topological signatures of the 4D h-HOTI are captured by the corresponding 1D array.

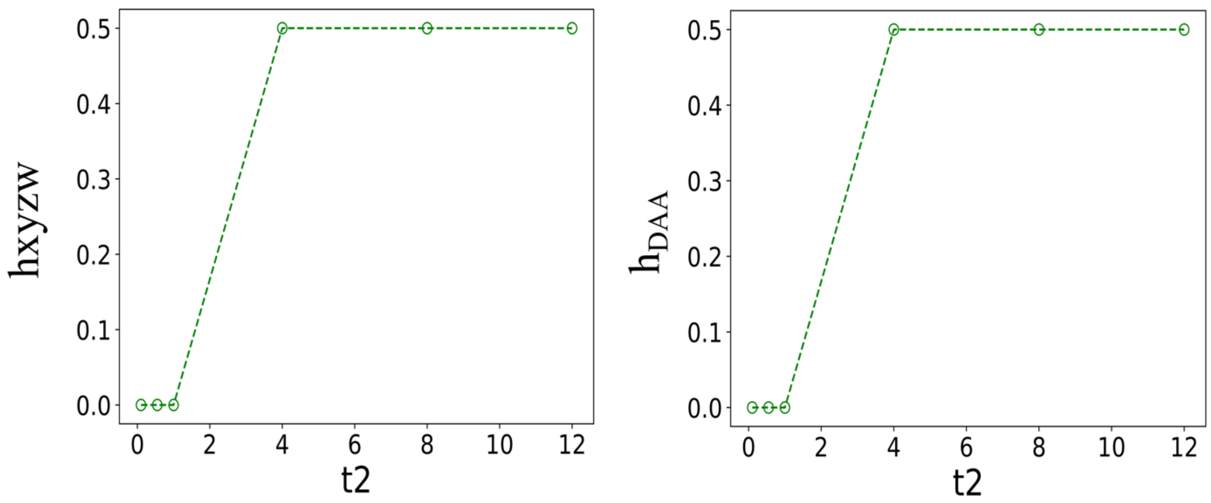

Fig. S6. Hexadecapole (h_{xyzw}) moments with intercell hopping amplitudes t2 for 4D h-HOTI and the corresponding topological invariant (h_{DAA}) for the effective 1D array. $N_x = N_y = N_z = N_w = 4$, intracell hopping amplitudes $t_1 = 1$.

S7: Band spectrum and amplitude distribution for 1D model with 30 sites.

As shown in Fig.S5, we calculated the band spectra and intensity distribution of the effective 1D model truncated after the 30th site, finding that the zero energy and the corresponding intensity

distributions of localized topological states are not affected, which confirm the feasibility of the dimensional reduction.

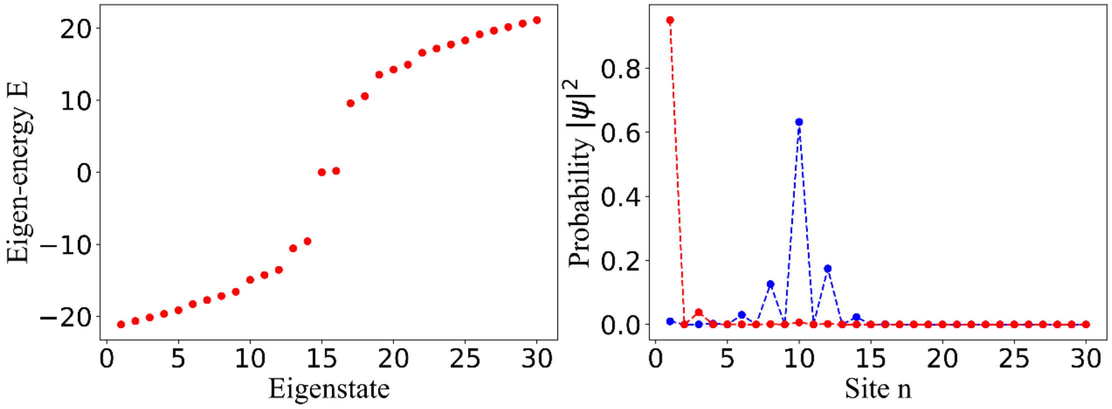

Fig. S7: (Left) Band spectra of the effective 1D model truncated to 30 sites obtained using the tight-binding model. (Right) The amplitude distributions corresponding to the projected 4th order topological corner mode in 1D array as found from tight-binding calculation.

S8: Hopping amplitude of the experiment.

As shown in Fig. S8, we use Comsol to simulate two coupled resonators and change the position of the connector ($dH = t * H_0$), the coupling equation is

$$\begin{bmatrix} \omega_0 & \gamma(t) \\ \gamma(t) & \omega_0 \end{bmatrix} \begin{bmatrix} \phi_1 \\ \phi_2 \end{bmatrix} = \omega \begin{bmatrix} \phi_1 \\ \phi_2 \end{bmatrix} \qquad (25)$$

By solving this simple equation, we get the spectrum $\omega_{\pm} = \omega_0 \pm \gamma(t)$, where $\omega_0 = \frac{v}{2\,H_0}$, v is the speed of sound. Then, we fit the spectrum data from Comsol (Fig. S8) and get the coupling as a function of t, the equation is $\gamma(t) = 301\left(t - \frac{1}{12}\right) + 7$. Finally, we can get coupling strength as a function of dH

$$\gamma(t) = \gamma(dH) = 301\left(\frac{dH}{H_0} - \frac{1}{12}\right) + 7 \qquad (26)$$

By using the above equation, we can map the coupling amplitude of our effective 1D chain to the distance dH of our experiment. The experiment data is shown in Table.1. In this paper, we focus on the first 20 sites of 1D chain, and each resonator has the same onsite energy. Therefore, these resonators can be set at the same height H_0.

dH[mm]	3	7.8	3	9.72
4.2	9	5.4	10.5	4.1
5.8	11.4	3.8	11.8	3.7
12	4.2	12	4.6	12

Table.1 The distances dH between the connectors and the middle point of the resonators.

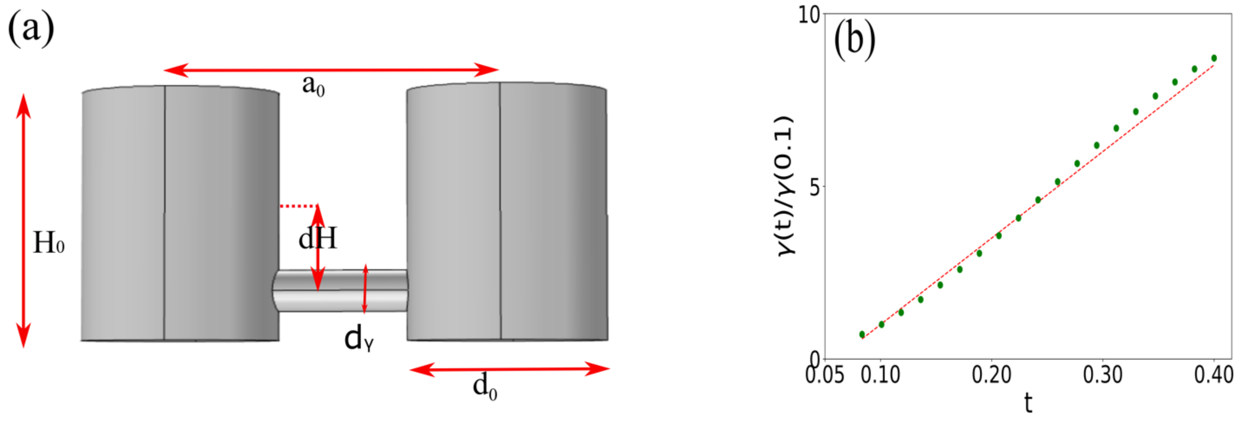

Fig. S8:. Schematics of the structure. (a) Two coupled resonators. The height of the cylinder is $H_0 = 30mm$, the diameter is $d_0 = 20mm$, the diameter of connector is $d_\gamma = 5mm$ and the distance between two resonators is $a_0 = 33mm$. (b) The coupling strength $\gamma(t)$ in the unit of $\gamma(0.1)$ as function of t, $\gamma(0.1)$ is the coupling strength between first and second resonator. Blue points are calculated by COMSOL and red dash line represents the line of fit.

S9: Quality factor

Two major loss mechanisms in our experiment are the absorption in the resin used in 3D printing and the leakage through the probe holes, which are deliberately introduced in the design of the individual resonators to allow excitation and probing of the acoustic field.

$$P(\omega) \propto \frac{1}{(\omega-\Omega)^2-\left(\frac{\Gamma}{2}\right)^2} \quad (27)$$

Where Ω is the resonance frequency and Γ is the linewidth of the resonance. We measure the frequency distribution with different heights of the resonator (See Fig. S9). By using equation (27) and quality factor $Q = \frac{\Omega}{\Gamma}$, we get the quality factor within the range of 50 to 60, and therefore, the corresponding finite lifetimes of the modes of the lattice are long enough not to alter their topological nature, making them clearly observable in our 1D array.

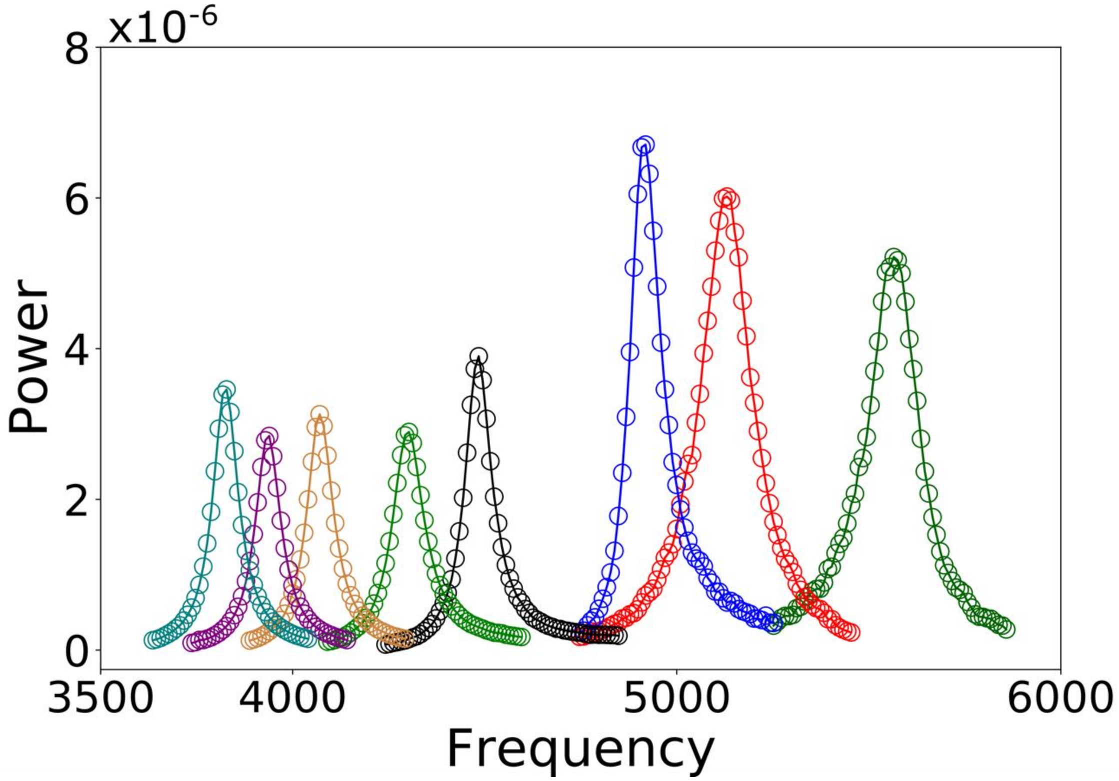

Fig. S9: Frequency distribution with heights $H_0 \in [30mm, 45mm]$.

References

1. Benalcazar WA, Bernevig BA, & Hughes TL (2017) Electric multipole moments, topological multipole moment pumping, and chiral hinge states in crystalline insulators. *Phys Rev B* 96(24).
2. Benalcazar WA, Bernevig BA, & Hughes TL (2017) Quantized electric multipole insulators. *Science* 357(6346):61-66.
3. Li X, Li X, & Sarma SD (2017) Mobility edges in one-dimensional bichromatic incommensurate potentials. *Physical Review B* 96(8):085119.
4. Agarwala A, Juričić V, & Roy B (2020) Higher-order topological insulators in amorphous solids. *Physical Review Research* 2(1):012067.
